# Factors Correlating with Functional Capacity in Older People with Chronic Pain

**DOI:** 10.3390/ijerph20032748

**Published:** 2023-02-03

**Authors:** Grażyna Puto, Iwona Repka, Marta Muszalik

**Affiliations:** 1 Institute of Nursing and Midwifery, Faculty of Health Sciences, Jagiellonian University Medical College, Kopernika 25 Street, 31-501 Krakow, Poland; 2 Department of Geriatrics, Faculty of Health Sciences, Nicolaus Copernicus University in Torun, Collegium Medicum in Bydgoszcz, 85-094 Bydgoszcz, Poland

**Keywords:** functional capacity, chronic pain, older people

## Abstract

Introduction: Chronic pain in older people is a global health problem not only in terms of a negative subjective feeling, but also as a social and economic factor. Deterioration of functional capacity is one of the main symptoms of chronic pain; therefore, it should be assessed as a basic parameter in the life of older people. The aim of the study was to analyze the factors which have an impact on the functional capacity of older people with chronic pain. Material and methods: The study was conducted among 181 people over 65 suffering from chronic pain lasting more than 6 months. The study used a questionnaire that included questions about demographic and social characteristics and the following scales: Abbreviated Mental Score (AMTS), Personal Activities of Daily Living (PADL) by Katz, Instrumental Activities of Daily Living (IADL) by Lawton, Geriatric Pain Measure-24 (GPM-24). Results: In the study group, a positive correlation was found between: coexisting diseases and withdrawal due to pain, pain intensity, pain resulting from walking or from the effort from other activities, and in terms of the total GPM-24 score. A positive correlation was also found between the Geriatric Depression Scale (GDS) and withdrawal due to pain, pain intensity, pain resulting from walking or from the effort from other activities, and in terms of the total GPM-24 score. A significantly negative correlation was found between: AMTS, ADL, IADL performance and: withdrawal due to pain, pain intensity, pain resulting from walking or from the effort from other activities, and in terms of total GPM-24 score. Conclusions: Chronic pain is more common in people with disabilities in basic and complex activities of daily living, with limited efficiency in cognitive functions and an increased sense of depression. The standard in everyday practice and clinical trials should be taking a history of chronic pain in every older person, monitoring the pain’s intensity and accompanying characteristics by using a multidimensional scale for assessing pain in older people.

## 1. Introduction

Chronic pain in older people is a global health problem not only in terms of a negative subjective feeling [1], but also as a social and economic factor. Currently, pain is defined as “an unpleasant sensory and emotional experience associated with actual or potential tissue damage, or described in terms of such damage” [1]. The intensity of pain and its duration have a significant impact on all areas of functioning of the people experiencing it. Chronic pain defined as pain that is felt for three to six months or more [2,3,4] is one of the most common ailments reported by older people and its incidence increases with age [3,5,6,7]. The prevalence of chronic pain among older people ranges from 25% to 75% of those living in the community [7,8,9] and up to 83% in long-term care facilities [2,7,8]. Pain is not a feature of physiological aging. Changes occurring in the aging process increase susceptibility to pain as well as affecting the ability to tolerate pain [5,6].

The older population is particularly vulnerable to the consequences of chronic pain. Since the deterioration of functional capacity is one of the main symptoms of chronic pain, it should be assessed as a basic parameter of life for older people. Functional capacity means the degree of physical fitness, the ability to independently perform certain activities indicating not only physical and mental condition, but also the degree of independence of an older person from the environment. This includes activities related to self-care, mobility, independence in performing basic daily activities that meet the main needs of the person [5,6,10]. Pain can limit movement, contributing to both loss of overall physical and mental performance [3,4,10,11], as well as to the occurrence of complications in the form of post-fall injuries [12,13,14]. The experience of chronic pain can be compounded by the accumulation of many potentially painful conditions [3,6]; it causes not only physical suffering but also emotional and psychosocial reactions [4,15]. Loss of appetite, depression [16,17] and social withdrawal are common reactions to painful conditions. Pain perception and pain responses can be modified by demographic, social, cultural factors and previous pain experience, as well as accompanying circumstances [18,19]. Long-term pain is a risk factor for mortality in the older people [7], which can lead to serious consequences such as cognitive impairment [6,7], memory, concentration, attention and sleep disorders [14], and can pose a risk of addiction to the medication [7]. The assessment and pharmacological treatment of chronic pain in the older people presents many challenges, is usually only partially effective and is often limited by comorbidities and adverse drug reactions. A comprehensive assessment of chronic pain is a prerequisite for adopting an interdisciplinary, holistic approach in order to provide older people with the most effective treatment that can significantly improve their quality of life [6,7,20]. The aim of the study was to analyze the factors which have an impact on the functional capacity of older people with chronic pain.

## 2. Materials and Methods

### 2.1. Data Collection

The study was conducted among people hospitalized in 4 medical treatment wards in hospitals in southern Poland. Before starting the research, an initial pilot study was carried out, the purpose of which was to verify the accuracy of the tools used. Data for the study were collected in 2016–2018 after analyzing medical records.

The analysis of medical records was aimed at getting acquainted with the medical diagnosis(es) and obtaining information on the presence of pain, its duration and severity (selection of the study group). The criterion for inclusion in the study was: age over 65, presence of pain > 6 months, no diagnosed cancer, no cognitive impairment (>3 AMTS points), any diseases being in their stable period (e.g., without dyspnea), obtaining written consent to participate in the study. The criteria for exclusion from the study were: age below 65, presence of pain < 6 months, diagnosed cancer, cognitive impairment (<3 AMTS points), unstable period of a disease, lack of written consent to participate in the study.

After obtaining the written consent of the patient to conduct the study, the patients (who met the criteria for inclusion in the study) were informed about the purpose of the study, as well as the possibility of asking questions and resigning from participation at any stage. The interview was conducted by the head or member of the research team on the premises of the hospital, ensuring privacy. The task of each respondent was to choose one answer from the options assigned to a given question. The duration of the test was approximately 15 min. Due to the need to access the documentation, the research was not anonymous. The results were encrypted, making it impossible to recognize the subject. The collected data were collected in an MS Office Excel spreadsheet and processed using statistical analysis.

### 2.2. Instruments

#### 2.2.1. General Characteristics

The study used a questionnaire that included questions about demographic and social characteristics (age, sex, education, place of residence, marital status, structure of residence) and assessment of the clinical condition (AMTS, ADL Katz, Lawton’s Scale, GPM-24, GDS, number of comorbidities).

#### 2.2.2. Geriatric Pain Measure-24

Pain was assessed using the Geriatric Pain Measure-24 (GPM-24) multidimensional scale developed by Bruce A. Ferrell et al. [21]. The psychometric properties of the Polish version of the scale were assessed by Puto et al. [22].

The GPM-24 multidimensional scale for assessing pain in older people consists of five subscales: disengagement because of pain (items 6, 17, 18, 19, 20, 21, 24), pain intensity (items 1, 2, 3, 4, 5, 22, 23), pain with ambulation (items 9, 10, 11, 12), pain with strenuous activities (items 8, 13, 14), and pain with other activities (items 7, 15, 16, 17, 22). Two scale items (17, 22) were included in two different subdimensions at the same time. GPM-24 contains 22 dichotomous questions with “yes/no” answers and 2 questions assessing the intensity of pain on a scale of 0–10. The total score (0–42) is obtained by summing each “yes” answer (1 point) together with the assessment of pain intensity (0–10 points). Adjusted Score (0–100) is the total score multiplied by 2.38. In the assessment of the GPM, a score below 30 indicates mild pain, a score of 30 to 69 indicates moderate pain, and a score greater than or equal to 70 indicates severe pain [21,22,23,24,25]. The Cronbach’s alpha reliability coefficient for the total score of the Polish version of the scale was 0.83 [21].

#### 2.2.3. Abbreviated Mental Test Score

The assessment of cognitive functions was carried out using the Abbreviated Mental Score (AMTS) scale intended for screening assessment—episodic, semantic and working memory of the older people. The scale consists of 10 items—questions and instructions for the tested person.

It should be noted that this is a screening tool. A positive result excludes dementia, and a negative result does not allow for a diagnosis but authorizes neuropsychological tests to be performed [26].

#### 2.2.4. ADL Katz Scale

Functional performance in basic activities of daily living (Personal Activities of Daily Living—PADL) was assessed using the ADL Katz scale. The scale enables the assessment of independent performance of activities, i.e., bathing the whole body, getting dressed and undressed, using the toilet, getting out of bed and moving to an armchair, controlling urine and stool excretion, eating meals independently [27].

#### 2.2.5. Lawton Scale

Functional ability to perform complex activities of daily living (Instrumental Activities of Daily Living—IADL), which includes: using the telephone, moving outside the home, preparing meals, doing household chores (cleaning, repairs, laundry, taking medications) as well as managing the budget, was carried out using the Lawton scale. The questions have assigned answers along with the score, i.e., the person is able to perform a task: without assistance (3 points, independent person), with a little help (2 points); unable to perform (1 point—depends on the environment). With a decrease in the number of points, a deterioration in functional efficiency is recognized [28].

#### 2.2.6. Geriatric Depression Scale

Feeling of depression was assessed using Geriatric Depression Screening (GDS-15). The questions have assigned answers of “yes/no” and are scored according to the scale scoring key [29,30].

### 2.3. Statistical Analysis

Distributions of qualitative variables were described using the absolute number of individual categories (N) and their percentage share in the distribution of the variable (%). The average values of quantitative variables were described using the mean and standard deviation (SD). The strength of the association between quantitatively measured variables was assessed using Spearman’s Rho coefficient. Relationships between qualitative variables were presented in the form of cross tables. The analysis of statistical significance of these relationships was performed using Pearson’s chi2 test (*p* ^chi2^). Comparison of mean values of normally distributed variables in two unrelated groups was performed using the Student’s *t*-test (*p* ^t^) for independent groups, and in more than two unrelated groups using ANOVA (*p* ^A^); effects for which *p* < 0.05 were considered statistically significant. Calculations were made using IBM SPSS Statistics 27 for Windows (IBM Corp. Released 2020. IBM SPSS Statistics for Windows, Version 27.0. Armonk, NY, USA: IBM Corp.).

### 2.4. Ethical Procedures

The study was conducted in accordance with the principles of the Declaration of Helsinki [31]. All collected data were stored in protected files and made available in accordance with the provisions of the General Data Protection Regulation [32,33]. The study was conducted after obtaining the consent of the Bioethics Committee KBET/83/B/2013.

### 2.5. Participants

Initially, 305 patients were recruited who reported pain of varying severity. However, 94 (34%) of them did not meet the above-mentioned criteria for inclusion in the study after analyzing their medical records (i.e., documented cancer or cognitive impairment) and 30 (9.8%) patients refused to participate in the study. The analysis covered 181 (59%) older people.

## 3. Results

### 3.1. Demographic and Social Characteristics of the Study Group

Among 181 people over 65 years of age, the percentage of women surveyed was higher than that of men (61.9% vs. 38.1%). The mean age in the study group was 77.1 (±7.9) years. There were 86 (47.5%) people in the study group with secondary education, 39 (21.5%) with higher education, 36 (20%) with vocational education, and 20 (11%) with primary education. As for the place of living, 127 (70.2%) people lived in the city, 54 (29.8%) in the countryside. Those who lost their partner as a result of his death and were divorced constituted a group of 100 (55.2%) persons, and 81 (44.8%) persons remained married. The structure of residence showed that 109 (60%) people lived with a family, 36 (20%) with a spouse and 36 (20%) alone.

### 3.2. Clinical Status of the Study Group

The assessment of cognitive functions carried out in the study group using the AMTS scale showed normal functioning in 161 (89%) of the examined subjects, while among 20 (11%) people, moderate impairment in cognitive functions was detected. The analysis of the degree of depression carried out according to the GDS showed a moderate sense of depression in 97 people (54%), a severe sense of depression in 19 people, and a normal result in 65 (36%) people. Functional capacity in terms of P-ADL assessed using the Katz scale showed that 137 (75.5%) people were able-bodied, 26 people (14.3%) moderately disabled, and 18 (10%) people suffered from a more profound disability.

The number of comorbidities (on average) in the study group was 4.1 ± 2.4. while in the group of disabled people in the range of IADL 19.1 ± 4.9. People with moderate cognitive impairment had a higher number of comorbidities than those functioning normally (6.2 ± 2.5 vs. 3.9 ± 2.3, *p* < 0.001). People with severe depression had the highest number of comorbidities, while the lowest number was found in those not suffering from depression (5.8 ± 2.0 vs. 3.4 ± 2.3, *p* < 0.001)—Table 1.

### 3.3. Demographic and Social Determinants of Pain in the Study Group

Women scored on average higher than men on: pain withdrawal (5.2 ± 1.9 vs. 4.5 ± 2.2, *p* = 0.03), pain intensity (15.8 ± 3.3 vs. 14.7 ± 3.6, *p* = 0.04), as well as the total (17.5 ± 4.8 vs. 15.8 ± 5.5, *p* = 0.03) and final (41.7 ± 11.5 vs. 36.3 ± 13.7, *p* = 0.006) of the GPM-24 score. More intense pain (moderate pain) was experienced by women more often than men (83.9% vs. 66.7%, *p* = 0.01).

Pain resulting from exertion—the highest score was found in people over 85 years of age, the lowest in the youngest age group (65–69 years old 2.9 ± 0.3 vs. 2.2 ± 1.0, *p* ˂ 0.001).

People with primary education obtained a higher score than people with secondary education in terms of: withdrawal due to pain (5.6 ± 1.8 vs. 4.3 ± 2.3, *p* = 0.003), pain intensity (16.5 ± 4.5 vs. 14.3 ± 2.9, *p* = 0.03), pain caused by exercise (2.8 ± 0.7 vs. 2.4 ± 1.0, *p* = 0.002) or other activities (3.1 ± 1.5 vs. 2.4 ± 1.5, *p* = 0.03). The final total score of the GPM-24 scale was the highest among people with vocational education and the lowest among people with higher education (44.8 ± 11.3 vs. 36.4 ± 14.3, *p* = 0.002).

A higher value of pain associated with walking was experienced by people living in the city than in the countryside (2.7 ± 1.5 vs. 2.2 ± 1.5, *p* = 0.04).

Single persons scored higher than people who had partners in terms of: withdrawal due to pain (5.3 ± 1.7 vs. 4.4 ± 2.2, *p* = 0.002), pain associated with walking (2.8 ± 1.5 vs. 2.6 ± 1.5, *p* = 0.02), exercise (2.7 ± 0.7 vs. 2.3 ± 1.0, *p* = 0.005) as well as total (17.9 ± 4.7 vs. 15.6 ± 5.4, *p* = 0.002) and final (42.2 ± 11.6 vs. 36.5 ± 13.1, *p* = 0.002) GPM-24 score. Pain of greater intensity (moderate pain) was more often experienced by single persons than by persons who had partners (84.0% vs. 69.1%, *p* = 0.02).

The analysis of the structure of residence showed that people living alone had a higher final total GPM-24 score than people living with a family (42.3 ± 13.3 vs. 39.9 ± 12.9, *p* = 0.04)—Table 2.

### 3.4. Functional Capacity of the Study Group

People with moderate cognitive impairment scored higher than those with no impairment in terms of: withdrawal due to pain (6.2 ± 0.9 vs. 4.8 ± 2.0, *p* = 0.001), pain intensity (17.0 ± 0.9 vs. 15.1 ± 3.4, *p* = 0.02), pain associated with walking (3.8 ± 0.9 vs. 2.4 ± 1.5, *p* < 0.001), pain caused by exercise (3.0 ± 0.0 vs. 2.5 ± 0.9, *p* = 0.006) or other actions (4.0 ± 1.3 vs. 2.5 ± 1.4, *p* < 0.001) as well as in respect to total (21.8 ± 2.3 vs. 16.2 ± 5.0, *p* < 0.001) and final (51.9 ± 5.5 vs. 38.1 ± 12.5, *p* < 0.001) GPM-24 score. More intense pain (moderate pain) was experienced by all moderately cognitively impaired subjects (100%) and the majority of normal subjects (75%).

People with severe depression scored higher than those with a normal state in terms of: withdrawal due to pain (6.3 ± 0.8 vs. 3.9 ± 2.0, *p* < 0.001), pain intensity (17.4 ± 3.3 vs. 14.5 ± 3.4, *p* < 0.001), pain related to walking (3.7 ± 1.0 vs. 2.0 ± 1.6, *p* < 0.001), pain caused by exercise (3.0 ± 0.0 vs. 2.4 ± 0.9, *p* = 0.002 or other actions (3.7 ± 1.0 vs. 2.0 ± 1.2, *p* < 0.001) as well as in respect to total (21.2 ± 2.0 vs. 14.5 ± 5.3, *p* < 0.001) and final (48.1 ± 11.0 vs. 33.9 ± 12.8, *p* < 0.001) GPM-24 score. Pain of greater intensity (moderate pain) was most often experienced by people with a severe (94.7%) and moderate (85.6%) sense of depression (*p* < 0.001).

People with disabilities in the P-ADL range scored higher than non-disabled people in terms of: withdrawal due to pain (6.7 ± 0.6 vs. 4.5 ± 2.0, *p* < 0.001), pain intensity (18.3 ± 3, 2 vs. 14.8 ± 3.3, *p* < 0.001), walking-related pain (3.4 ± 1.3 vs. 2.3 ± 1.6, *p* = 0.001), pain caused by exercise (2.8 ± 0.7 vs. 2.5 ± 0.9, *p* = 0.002) or other actions (4.3 ± 0.9 vs. 2.3 ± 1.4, *p* < 0.001) as well as in respect to total (21.8 ± 2.8 vs. 15.7 ± 5.1, *p* < 0.001) and final (51.9 ± 6.8 vs. 36.9 ± 12.6, *p* < 0.001) GPM-24 score. Pain of greater intensity (moderate pain) was experienced by all disabled (100%) and partially functional (100%) patients in the PADL range, and mild pain (29.9%) was experienced by non-disabled people (*p* < 0.001).

The number of comorbidities was higher among those experiencing more intense pain (moderate pain) than among those experiencing less intense pain (mild pain) (4.41 ± 2.5 vs. 3.2 ± 1.9, *p* = 0.001). People with greater disability in the IADL scale experienced pain of greater intensity (moderate pain) than those with less disability (mild pain) (17.93 ± 4.5 vs. 23.26 ± 3.6, *p* < 0.001)—Table 3.

The analysis of Spearman’s Rho coefficient in the study group showed a significantly positive correlation between:

- The number of comorbidities and: withdrawal due to pain, intensity of pain, pain resulting from walking, effort from undertaking other activities, and in terms of total and final GPM-24 score;
- GDS a: withdrawal due to pain, pain intensity, pain resulting from: walking, effort during other activities, and total and final GPM-24 score;
- Significantly negative correlation between:
- Performance in AMTS, ADL, IADL and: withdrawal due to pain, pain intensity, pain resulting from: walking, effort from other activities, and in terms of the total and final score of the GPM-24 scale—Table 4.

## 4. Discussion

The paper presents the results of the first study using the GPM-24 scale after adaptation to Polish cultural conditions. There are few reports in the literature on the assessment of chronic pain using the GPM-24. Most of the studies conducted so far are selective and concern the assessment of psychometric properties of the GPM-24 [21,22,23,24,25,34,35]. There are no results that would present the analysis of factors determining the functional efficiency of older people with chronic pain assessed in the GPM-24.

In the Polish study, as well as in the study by the authors of the GPM-24 scale [21], higher pain scores were confirmed in women than in men. Women experienced more moderate pain than men. The Polish study also showed an increase in the intensity of pain with age—after the age of 85, moderate pain was reported by 91.2% of the respondents. The German study showed the highest frequency of pain and differences in pain intensity between women and men among people aged 85–89 [36]. Gender differences in pain intensity have been documented in other studies. Women more often than men experience pain of greater intensity [7,9,36,37]. The PolSenior2 study conducted among 5947 older people also confirmed a higher incidence of pain among women. The incidence of pain increased with age. The highest percentage of people reporting pain was in the age group of 85 to 89 (62.2%). In this study, pain intensity (average) was 6 points on the VAS scale, which corresponded to moderate pain intensity and was significantly higher in women [9]. In our study, pain intensity assessed with the GPM-24 scale was moderate and occurred more often in women. The demographic and social variables related to chronic pain in the study were: level of education, place of residence, structure of residence, marital status. People with primary education showed the highest score on experienced pain. People living in the city had the highest score for walking pain. Marital status showed a relationship with pain in single people. The relationship between the level of education and marital status was confirmed in a study conducted in the United States, where people with and without pain were compared. Chronic pain was more common among people with a lower level of education and among people who were divorced, separated, or never married than among people who were married or living with a partner [37]. In the surveyed population of PolSenior2, chronic pain was more often reported by people with primary education, living in rural areas and people living alone [9]. Our study confirmed that people living alone experienced pain more often than people living with a spouse/partner or family. Social support from a family member or caregiver can clearly influence the perception of pain in older people, which was confirmed in a study by Gallant and Hadjistavropoulos (2017). This study showed that the presence of a caregiver reduces pain compared to people living alone [38].

Both in our research and in the research of other authors [9], disability was associated with the presence of chronic pain. The relationship between functional ability and: withdrawal due to pain, pain intensity, pain associated with walking, resulting from exercise or from undertaking other activities, and in terms of the total and final score of the GPM-24 scale, was demonstrated in our study. Severely and moderately disabled patients as assessed by the PADL scale experienced pain of greater intensity (moderate pain). Disability was associated with chronic pain in another study. People who were moderately disabled as per the ADL reported pain most often, and non-disabled people least often. Partial and complete disability in IADL function were associated with chronic pain. The most common form of disability is motor disability, which requires the use of assistive equipment when moving around. People using crutches/canes reported chronic pain more often than people in a wheelchair or lying down [9]. The impact of pain on the functional capacity of older people was confirmed in a study conducted in Brazil by Fernandes et al. (2022); people who reported pain presented a lower score in terms of walking speed, maintaining balance, obtaining a higher score in terms of fear of falling [12]. Falling as a common problem among older people living in the community was observed in a study by Kulakci Altintas and Korkmaz Aslan (2019), while pain (assessed by GPM-24) and insomnia were identified as risk factors for falls [14].

According to Zis et al. (2017), chronic pain and depression are common in the older people. The authors concluded that depression and pain may be mutual risk factors [39]. The relationship between depression and: withdrawal due to pain, pain intensity, pain resulting from: walking or from undertaking other activities, and in terms of the total and final score of the GPM-24 scale was confirmed in the study. The link between pain and depression was confirmed in a study by Lee et al. (2018) among older people. The results of these studies show that undiagnosed depression in people with chronic pain is common. The authors emphasize that pain clinicians should actively seek to identify the presence of depression among people with pain [40]. No relationship between depression and pain (assessed using the GPM-24 scale) was shown in a study conducted in England by Iliffe et al. (2009). The predictors of depression in this study were disability in basic activities of daily living, the risk of social isolation and primary education [41].

### Strengths and Limitations

It should be emphasized that the present study has some limitations. The conducted study was cross-sectional. Assessment of the functional capacity of people with pain would show better validity in longitudinal studies, making it a continuity parameter. Secondly, the limitation of the presented results is also the fact that the localization of pain, which could have a significant impact on functional efficiency, was not taken into account. Thirdly, the assessment of chronic pain is conditioned by self-esteem and cognitive functions of the older people. People with cognitive disorders were excluded from the study, which ensured greater reliability of the information obtained, limiting the possibility of obtaining results among people with those disorders. Comparing the obtained results is difficult due to the different way in which pain is defined and evaluated by other authors with one-dimensional scales. There are no results of studies assessing pain in a multidimensional way. The strength of the study, to the best of our knowledge, is that it is one of the first analyses to evaluate chronic pain using the multidimensional GPM-24 scale. This scale, recommended for older people suffering from many coexisting diseases, assesses the intensity of pain, its psychological and functional aspects, which are a key element of loss of independence, limitation of capacity and exclusion from social and spiritual life.

## 5. Conclusions

Chronic pain is more common in people with disabilities in basic and complex activities of daily living, with limited capacity in cognitive functions and an increased sense of depression. The standard in everyday practice and clinical trials should be taking a history of chronic pain in every older person, monitoring its intensity and accompanying characteristics using a multidimensional scale for assessing pain in the older people.

## Figures and Tables

**Table 1:** Clinical status conditioned by the number of coexisting diseases.

Variables		Clinical Condition		Number of Comorbidities	*p* ^t^
		N	%	Mean ± SD	
AMTS	Normal score (6–10 pts)	161	89	3.9 ± 2.3	<0.001
	Moderate impairment (4–5 pts)	20	11	6.2 ± 2.5	
GDS	Normal score (0–5 pts)	65	36	3.4 ± 2.3	<0.001
	Moderate depression (6–10 pts)	97	54	4.3 ± 2.4	
	Severe depression (11–15 pts)	19	10	5.8 ± 2.0	
PADL	Profound impairment (0–2 pts)	18	10	4.9 ± 2.8	0.09
	Moderate impairment (3–4 pts)	26	14.3	3.3 ± 2.0	
	Normal score (5–6 pts)	137	75.7	4.2 ± 2.4	
Number of comorbidities				4.1 ± 2.4	-
IADL by Lawton (range 0–27 pts)				19.1 ± 4.9	-

N—Number of subjects; %—Percentage of respondents; AMTS—Abbreviated Mental Score; GDS—Geriatric Depression Screening; PADL—Personal Activities of Daily Living; IADL—Instrumental Activities of Daily Living; Mean—Arithmetic mean; SD—Standard deviation; *p* ^t^ value—For Student’s *t*-test.

**Table 2:** Demographic and social variables determine the functional capacity of people with chronic pain.

Variables	GPM-24									
	Subscale					Score		Pain Intensity		
	Disengagement Because of Pain	Pain Intensity	Pain with Ambulation	Pain with Strenuous Activities	Pain with Other Activities	Total	Adjusted	<30 Mild Pain	30–69 Moderate Pain	>70 Severe Pain
	Mean ± SD	Mean ± SD	Mean ± SD	Mean ± SD	Mean ± SD	Mean ± SD	Mean ± SD	N (%)	N (%)	N (%)
Sex										
women	5.2 ± 1.9	15.8 ± 3.3	2.7 ± 1.5	2.6 ± 0.8	2.8 ± 1.4	17.5 ± 4.8	41.7 ± 11.5	18 (16.1)	94 (83.9)	0 (0.0)
men	4.5 ± 2.2	14.7 ± 3.6	2.4 ± 1.6	2.5 ± 0.9	2.4 ± 1.5	15.8 ± 5.5	36.3 ± 13.7	23 (33.3)	46 (66.7)	0 (0.0)
*p* ^t^	0.03	0.04	0.25	0.38	0.05	0.03	0.006	*p* ^Chi2^ = 0.01		
Age (yrs)										
65–69	4.6 ± 2.2	14.6 ± 2.8	2.0 ± 1.7	2.2 ± 1.0	2.1 ± 1.5	14.9 ± 5.4	35.5 ± 13.0	15 (34.1)	29 (65.9)	0 (0.0)
70–74	5.0 ± 2.0	15.2 ± 3.5	2.6 ± 1.5	2.7 ± 0.6	2.9 ± 1.4	17.4 ± 4.8	41.4 ± 11.4	5 (17.2)	24 (82.8)	0 (0.0)
75–79	4.7 ± 2.2	15.4 ± 3.8	2.8 ± 1.4	2.4 ± 1.0	2.7 ± 1.4	16.9 ± 5.3	38.9 ± 13.7	10 (27.8)	26 (72.2)	0 (0.0)
80–84	5.2 ± 1.9	16.0 ± 3.6	2.7 ± 1.4	2.6 ± 0.8	2.9 ± 1.5	17.6 ± 5.3	40.8 ± 13.7	8 (21.1)	30 (78.9)	0 (0.0)
85 and over	5.2 ± 1.6	15.8 ± 3.6	2.8 ± 1.4	2.9 ± 0.3	2.8 ± 1.4	18.0 ± 4.1	43.2 ± 9.7	3 (8.8)	31(91.2)	0 (0.0)
*p* ^A^	0.61	0.36	0.10	˂0.001	0.08	0.06	0.08	*p* ^Chi2^ = 0.09		
Education										
primary	5.6 ± 1.8	16.5 ± 4.5	3.0 ± 1.2	2.8 ± 0.7	3.1 ± 1.5	19.2 ± 3.9	43.5 ± 12.5	3 (15.0)	17 (85.0)	0 (0.0)
vocational	5.6 ± 1.7	15.7 ± 3.1	3.1 ± 1.4	2.8 ± 0.4	3.0 ± 1.6	18.9 ± 4.7	44.8 ± 11.3	4 (11.1)	32 (88.9)	0 (0.0)
secondary	4.7 ± 1.9	15.4 ± 3.5	2.2 ± 1.5	2.4 ± 1.0	2.5 ± 1.4	15.9 ± 4.9	38.0 ± 11.7	21 (24.4)	65 (75.6)	0 (0.0)
higher	4.3 ± 2.3	14.3 ± 2.9	2.6 ± 1.5	2.4 ± 1.0	2.4 ± 1.5	15.8 ± 5.7	36.4 ± 14.3	13 (33.3)	26 (66.7)	0 (0.0)
*p* ^A^	0.003	0.03	0.05	0.002	0.03	0.001	0.002	*p* ^Chi2^ = 0.10		
Place of living										
city	5.0 ± 2.0	15.4 ± 3.3	2.7 ± 1.5	2.6 ± 0.8	2.7 ± 1.5	17.2 ± 5.0	40.4 ± 12.6	27 (21.3)	100 (78.7)	0 (0.0)
countryside	4.6 ± 2.0	15.4 ± 3.8	2.2 ± 1.5	2.5 ± 0.9	2.5 ± 1.3	16.0 ± 5.3	38.0 ± 12.7	14 (25.9)	40 (74.1)	0 (0.0)
*p* ^t^	0.15	0.76	0.04	0.63	0.42	0.15	0.25	*p* ^Chi2^ = 0.56		
Marital status										
in a relationship	4.4 ± 2.2	15.0 ± 3.7	2.6 ± 1.5	2.3 ± 1.0	2.4 ± 1.3	15.6 ± 5.4	36.5 ± 13.1	25 (30.9)	56 (69.1)	0 (0.0)
single	5.3 ± 1.7	15.7 ± 3.3	2.8 ± 1.5	2.7 ± 0.7	2.8 ± 1.5	17.9 ± 4.7	42.2 ± 11.6	16 (16.0)	84 (84.0)	0 (0.0)
*p* ^t^	0.002	0.18	0.02	0.005	0.09	0.002	0.002	*p* ^Chi2^ = 0.02		
Residence structure										
alone	5.4 ± 1.9	15.8 ± 3.0	2.9 ± 1.6	2.5 ± 1.0	2.8 ± 1.7	17.8 ± 5.5	42.3 ± 13.2	7 (19.4)	29 (80.6)	0 (0.0)
with a partner	4.8 ± 2.0	15.6 ± 3.4	2.4 ± 1.4	2.4 ± 0.9	2.5 ± 1.4	16.4 ± 4.7	39.1 ± 11.2	7 (19.4)	29 (80.6)	0 (0.0)
with familty	4.8 ± 2.0	15.2 ± 3.6	2.5 ± 1.5	2.6 ± 1.0	2.6 ± 1.4	16.7 ± 5.1	39.0 ± 12.9	27 (24.8)	82 (75.2)	0 (0.0)
*p* ^A^	0.27	0.57	0.38	0.57	0.72	0.47	0.04	*p* ^Chi2^ = 0.70		

N—number of subjects; %—percentage of respondents; GPM-24—Geriatric Pain Measure-24; Mean—arithmetic mean; SD—standard deviation; value: *p* ^A^—for ANOVA test; *p* ^Chi2^—for Chi2 test; *p* ^t^—for Student’s *t*-test.

**Table 3:** Functional capacity variables correlate with pain measures in people with chronic pain.

Variables	GPM-24									
	Subscale					Score		Pain Intensity		
	Disengagement Because of Pain	Pain Intensity	Pain with Ambulation	Pain with Strenuous Activities	Pain with Other Activities	Total	Adjusted	<30 Mild Pain	30–69 Moderate Pain	>70 Severe Pain
	Mean ± SD	Mean ± SD	Mean ± SD	Mean ± SD	Mean ± SD	Mean ± SD	Mean ± SD	N (%)	N (%)	N (%)
AMTS										
Normal score	4.8 ± 2.0	15.1 ± 3.4	2.4 ± 1.5	2.5 ± 0.9	2.5 ± 1.4	16.2 ± 5.0	38.1 ± 12.5	41 (25.5)	120 (74.5)	0 (0.0)
Moderate impairment	6.2 ± 0.9	17.0 ± 3.2	3.8 ± 0.9	3.0 ± 0.0	4.0 ± 1.3	21.8 ± 2.3	51.9 ± 5.5	0 (0.0)	20 (100)	0 (0.0)
*p* ^t^	0.001	0.02	˂0.001	0.006	˂0.001	˂0.001	˂0.001	*p*^Chi2^ = 0.008		
GDS										
Normal score	3.9 ± 2.0	14.5 ± 3.4	2.0 ± 1.6	2.4 ± 0.9	2.0 ± 1.2	14.5 ± 5.3	33.9 ± 12.8	26 (40.0)	39 (60.0)	0 (0.0)
Moderate depression	5.3 ± 1.9	15.6 ± 3.3	2.7 ± 1.4	2.6 ± 0.8	2.8 ± 1.6	17.6 ± 4.7	41.8 ± 11.2	14 (14.4)	83 (85.6)	0 (0.0)
Severe depression	6.3 ± 0.8	17.4 ± 3.3	3.7 ± 1.0	3.0 ± 0.0	3.7 ± 1.0	21.2 ± 2.0	48.1 ± 11.0	1 (5.3)	18 (94.7)	0 (0.0)
*p* ^A^	˂0.001	˂0.001	˂0.001	0.002	˂0.001	˂0.001	˂0.001	*p*^Chi2^ ˂ 0.001		
P-ADL										
Normal score	4.5 ± 2.0	14.8 ± 3.3	2.3 ± 1.6	2.5 ± 0.9	2.3 ± 1.4	15.7 ± 5.1	36.9 ± 12.6	41 (29.9)	96 (70.1)	0 (0.0)
Moderate disability	5.9 ± 1.3	16.2 ± 3.5	3.0 ± 1.1	2.8 ± 0.6	3.1 ± 1.3	19.3 ± 3.2	45.9 ± 7.7	0 (0.0)	26 (100)	0 (0.0)
Severe disability	6.7 ± 0.6	18.3 ± 3.2	3.4 ± 1.3	2.8 ± 0.7	4.3 ± 0.9	21.8 ± 2.8	51.9 ± 6.8	0 (0.0)	18 (100)	0 (0.0)
*p* ^A^	˂0.001	˂0.001	0.001	0.002	˂0.001	˂0.001	˂0.001	*p*^Chi2^ ˂ 0.001		
								mean ± SD	mean ± SD	mean ± SD
Comorbidities								3.2 ± 1.9	4.4 ± 2.5	00.00
*p* ^t^								*p* = 0.001		
I-ADL the more points, the better the capacity								23.2 ± 3.6	17.9 ± 4.5	0.0 ± 0.0
*p* ^t^								*p* ˂ 0.001		

N—number of subjects; %—percentage of respondents; GPM-24—Geriatric Pain Measure-24; SD—standard deviation; value: *p* ^A^—for ANOVA test; *p* ^Chi2^—for Chi2 test; *p* ^t^—for Student’s *t*-test.

**Table 4:** Spearman’s Rho correlation of variables determining the functional capacity of people with chronic pain.

Variables		GPM-24						
		Subscale					Score	
		Disengagement Because of Pain	Pain Intensity	Pain with Ambulation	Pain with Strenuous Activities	Pain with Other Activities	Total	Adjusted
AMTS	rho	−0.36	−0.21	−0.29	−0.34	−0.31	−0.40	−0.39
	*p*	0.000	0.004	0.000	0.000	0.000	0.000	0.000
GDS	rho	0.46	0.25	0.35	0.24	0.39	0.45	0.45
	*p*	0.000	0.001	0.000	0.001	0.000	0.000	0.000
P-ADL	rho	−0.46	−0.32	−0.28	−0.27	−0.43	−0.45	−0.47
	*p*	0.000	0.000	0.000	0.000	0.000	0.000	0.000
I-ADL	rho	−0.51	−0.35	−0.39	−0.41	−0.51	−0.55	−0.55
	*p*	<0.001	<0.001	<0.001	<0.001	<0.001	<0.001	<0.001
Comorbidities	rho	0.16	0.23	0.40	0.15	0.29	0.30	0.29
	*p*	0.03	0.002	<0.001	0.05	<0.001	<0.001	<0.001

AMTS—Abbreviated Mental Score; GDS—Geriatric Depression Screening; PADL—Personal Activities of Daily Living; IADL—Instrumental Activities of Daily Living; GPM-24—Geriatric Pain Measure-24; *p* for Spearman’s Rho correlation.

## Data Availability

The data presented in this study are available on reasonable request from the corresponding author.

## References

[1] Raja S.N., Carr D.B., Cohen M., Finnerup N.B., Flor H., Gibson S., Keefe F.J., Mogil J.S., Ringkamp M., Sluka K.A. (2020). The revised International Association for the Study of Pain definition of pain: Concepts, challenges, and compromises. Pain.

[2] Cravello L., Di Santo S., Varrassi G., Benincasa D., Marchettini P., de Tommaso M., Shofany J., Assogna F., Perotta D., Palmer K. (2019). Chronic Pain in the Elderly with Cognitive Decline: A Narrative Review. Pain Ther..

[3] Dagnino A.P.A., Campos M.M. (2022). Chronic Pain in the Elderly: Mechanisms and Perspectives. Front. Hum. Neurosci..

[4] Treede R.D., Rief W., Barke A., Aziz Q., Bennett M.I., Benoliel R., Cohen M., Evers S., Finnerup N.B., First M.B. (2019). Chronic pain as a symptom or a disease: The IASP Classification of Chronic Pain for the International Classification of Diseases (ICD-11). Pain.

[5] Schofield P. (2018). The Assessment of Pain in Older People: UK National Guidelines. Age Ageing.

[6] Mullins S., Hosseini F., Gibson W., Thake M. (2022). Physiological changes from ageing regarding pain perception and its impact on pain management for older adults. Clin. Med..

[7] Domenichiello A.F., Ramsden C.E. (2019). The silent epidemic of chronic pain in older adults. Prog. Neuropsychopharmacol. Biol. Psychiatry.

[8] de Souza I.M.B., Sakaguchi T.F., Yuan S.L.K., Matsutani L.A., do Espírito-Santo A.S., Pereira C.A.B., Marques A.P. (2019). Prevalence of low back pain in the elderly population: A systematic review. Clinics.

[9] Kozak-Szkopek E., Kujawska-Danecka H., Wierzba K., Szybalska A., Mossakowska M., Błędowski P., Grodzicki T., Mossakowska M., Zdrojewski T. (2021). Występowanie bólu przewlekłego. Badanie Poszczególnych Obszarów Stanu Zdrowia Osób Starszych, w Tym Jakości Życia Związanej ze Zdrowiem.

[10] Dueñas M., Ojeda B., Salazar A., Mico J.A., Failde I. (2016). A review of chronic pain impact on patients, their social environment and the health care system. J. Pain Res..

[11] Lemos B.D.O., Cunha A.M.R.D., Cesarino C.B., Martins M.R.I. (2019). The impact of chronic pain on functionality and quality of life of the elderly. BrJP.

[12] Fernandes S.G., Sales W.B., Tavares D.V., Pereira D.d.S., Nóbrega P.V.d.N., Holanda C.M.d.A., Maciel A.C.C. (2022). Relationship between Pain, Fear of Falling and Physical Performance in Older People Residents in Long-Stay Institutions: A Cross-Sectional Study. Int. J. Environ. Res. Public Health.

[13] Welsh V.K., Clarson L.E., Mallen C.D., McBeth J. (2019). Multisite pain and self-reported falls in older people: Systematic review and meta-analysis. Arthritis Res..

[14] Kulakci Altintas H., Korkmaz Aslan G. (2019). Incidence of falls among community-dwelling older adults in Turkey and its relationship with pain and insomnia. Int. J. Nurs. Pract..

[15] Inoue S., Taguchi T., Yamashita T., Nakamura M., Ushida T. (2017). The prevalence and impact of chronic neuropathic pain on daily and social life: A nationwide study in a Japanese population. Eur. J. Pain.

[16] Lerman S.F., Rudich Z., Brill S., Shalev H., Shahar G. (2015). Longitudinal associations between depression, anxiety, pain, and pain-related disability in chronic pain patients. Psychosom. Med..

[17] Jones M.R., Ehrhardt K.P., Ripoll J.G., Sharma B., Padnos I.W., Kaye R.J., Kaye A.D. (2016). Pain in the elderly. Curr. Pain Headache Rep..

[18] Kosson D., Wordliczek J., Malec-Milewska M., Woroń J. (2017). Leczenie bólu u osób w wieku podeszłym. Kompendium Leczenia Bólu.

[19] Rosenthal T., Naughton B., Williams M.E., Pączek L., Niemczyk M. (2009). Geriatria.

[20] Ali A., Arif A.W., Bhan C., Kumar D., Malik M.B., Sayyed Z., Akhtar K.H., Ahmad M.Q. (2018). Managing Chronic Pain in the Elderly: An Overview of the Recent Therapeutic Advancements. Cureus.

[21] Ferrell B.A., Stein W.M., Beck J.C. (2000). The Geriatric Pain Measure: Validity, reliability and factor analysis. J. Am. Geriatr. Soc..

[22] Puto G., Repka I., Brzyski P. (2021). Pain measurement in the older people: Evaluation of the psychometric properties of the Geriatric Pain Measure (GPM-24)—Polish version. BMC Geriatr..

[23] Clough-Gorr K.M., Blozik E., Gillmann G., Beck J.C., Ferrell B.A., Anders J., Harari D., Stuck A.E. (2008). The self-administered 24-item geriatric pain measure (GPM-24-SA): Psychometric properties in three European populations of community-dwelling older adults. Pain Med..

[24] Streiner D.L., Norman G.R. (2008). Health Measurement Scales: A Practical Guide to Their Development and Use.

[25] Blozik E., Stuck A.E., Niemann S., Ferrell B.A., Harari D., von Renteln-Kruse W., Gillmann G., Beck J.C., Clough-Gorr K.M. (2007). Geriatric Pain Measure Short Form: Development and Initial Evaluation. J. Am. Geriatr. Soc..

[26] Romanik W., Łazarewicz M. (2017). Wersja polska Skróconego Testu Sprawności Umysłowej (AMTS)—Problemy metodologiczne. Psychiatr. Psychol. Klin..

[27] Katza S., Ford A.B., Moskowitz R.W., Jackson B.A., Jaffe M.W. (1963). Studies of illness in aged: The index of ADL: A standardized measure of biological and psychosocial function. JAMA.

[28] Lawton M.P., Brody E.M. (1969). Assessment of older people: Self-maintaining and instrumental activities of daily living. Gerontologist.

[29] Albiński R., Kleszczewska-Albińska A., Bedyńska S. (2011). Geriatryczna Skala Depresji (GDS). Trafność i rzetelność różnych wersji tego narzędzia—Przegląd badań. Psychiatr. Pol..

[30] Yesavage J.A., Brink T.L., Rose T.L., Lum O., Huang V., Adey M., Leirer V.O. (1983). Development and evaluation of a geriatric depression screening scale: A preliminary report. J. Psychiatr. Res..

[31] World Medical Association (2013). World Medical Association Declaration of Helsinki: Ethical principles for medical research involving human subjects. JAMA.

[32] European Parliament and of the Council (EU) Regulation of the European Parliament and of the Council (EU) 2016/679 of 27 April 2016 on the Protection of Individuals with Regard to the Processing of Personal Data and on the Free Movement of Such Data and the Repeal of Directive 95/46/EC (General Data Protection Regulation). https://eur-lex.europa.eu/legal-content/PL/TXT/?uri=CELEX%3A32016R0679.

[33] Chancellery of the Sejm of the Republic of Poland The Act of 10 May 2018 on the Protection of Personal Data. *J. Laws*
**2018**. https://uodo.gov.pl/en/594.

[34] Dursun G., Bektas H. (2017). Cultural Validation and Reliability of the Turkish Version of the Geriatric Pain Measure in the Elderly. Pain Pract..

[35] Motta T., Gambaro R., Santos F. (2015). Pain measurement in the elderly: Evaluation of psychometric properties of the Geriatric Pain Measure—Portuguese version. Rev. Dor..

[36] Mallon T., Ernst A., Brettschneider C., König H.H., Luck T., Röhr S., Weyerer S., Werle J., Mösch E., Weeg D. (2018). Prevalence of pain and its associated factors among the oldest-olds in different care settings—Results of the AgeQualiDe study. BMC Fam. Pract..

[37] Pitcher M.H., Von Korff M., Bushnell M.C., Porter L. (2019). Prevalence and Profile of High-Impact Chronic Pain in the United States. J. Pain.

[38] Gallant N.L., Hadjistavropoulos T. (2017). Experiencing Pain in the Presence of Others: A Structured Experimental Investigation of Older Adults. J. Pain.

[39] Zis P., Daskalaki A., Bountouni I., Sykioti P., Varrassi G., Paladini A. (2017). Depression and chronic pain in the elderly: Links and management challenges. Clin. Interv. Aging.

[40] Lee H.J., Choi E.J., Nahm F.S., Yoon I.Y., Lee P.B. (2018). Prevalence of unrecognized depression in patients with chronic pain without a history of psychiatric diseases. Korean J. Pain.

[41] Iliffe S., Kharicha K., Carmaciu C., Harari D., Swift C., Gillman G., Stuck A.E. (2009). The relationship between pain intensity and severity and depression in older people: Exploratory study. BMC Fam. Pract..

